# Gravitational effects on the hydrogen bond network of water and ionic solutions revealed by near infrared spectroscopy under simulated microgravity

**DOI:** 10.1038/s41598-026-44169-1

**Published:** 2026-03-14

**Authors:** Mika Ishigaki, Koyo Koizumi, Kotomi Asano, Naoki Okamoto, Go Takehi, Riku Sasamoto, Masato Takeuchi, Roumiana Tsenkova, Mio Matsui, Aiko Nagamatsu, Mariko Egawa

**Affiliations:** 1 Institute of Agricultural and Life Sciences, Shimane University, Matsue, Japan; 2 MIRAI Technology Institute, Shiseido Co., Ltd., Yokohama, Japan; 3 Department of Applied Chemistry, Graduate School of Engineering, Osaka Metropolitan University, Osaka, Japan; 4 Graduate School of Agricultural Science, Kobe University, Kobe, Japan; 5 Space Exploration Innovation Hub Center, Japan Aerospace Exploration Agency, Sagamihara, Japan

**Keywords:** Biophysics, Chemistry, Physics

## Abstract

The near-infrared spectra of ultrapure water and five types of ionic aqueous solutions were recorded under normal and simulated microgravity (< 0.1 G). The water band at approximately 1450 nm, corresponding to the combination of O–H symmetric and antisymmetric stretching vibrations, was analyzed. The results showed that the hydrogen-bond network (HBN) weakened in both ultrapure water and all ionic solutions under microgravity. This weakening is physically reasonable when considered in terms of a slight decrease in hydrostatic pressure leading to volumetric expansion of water. Furthermore, weakening of the HBN by gravity changes was smaller than that caused by modest temperature changes of approximately 2 °C and varied depending on the type of ions present. Specifically, gravity-induced changes in the HBN of water were less pronounced in solutions containing kosmotropic anions than in solutions containing chaotropic anions. These findings suggest that even subtle changes in HBN could disrupt finely tuned biochemical reactions, potentially influencing human health in altered gravity environments.

**Supplementary Information:**

The online version contains supplementary material available at 10.1038/s41598-026-44169-1.

## Introduction

Earth is known as the *water planet*, and water is essential for all living organisms. Water molecules form a complex network of hydrogen bonds, giving rise to anomalous properties—such as a high boiling point despite its low molecular weight and maximum density at 4°C^1,2^. Hydrogen bonding in liquid water is a fundamental property that enables water to support life. Water molecules are among the simplest and most abundant molecules in nature. However, their unusual properties are still not fully understood^3–5^. Under microgravity in space, the physical distances between water molecules will change, and the hydrogen-bond network (HBN) and intermolecular interactions of water molecules are expected to differ from those on Earth. As the prospect of human migration into space becomes realistic, we must seriously consider whether organisms, especially those with a high water content, can maintain healthy physiological functions in space. If the dynamics of water molecules in space differ from that on Earth, biochemical reactions in the body could be altered, potentially affecting human health. Therefore, the aim of this study was to address a fundamental question: How might the dynamics of water molecules in microgravitational environments differ from those on Earth?

Several studies have examined the dynamics of water, ionic solutions, and hydrogels in space or under microgravity. Noble et al. used a pulsed monochromatic mid-IR laser to induce changes in the hydrogen bonding of amorphous solid water (ASW) present in the interstellar medium of the solar system^6^. They found that resonant irradiation increases ASM crystalline-like structures while decreasing ASM amorphous structures. Matsushima et al. analyzed water electrolysis in both alkaline and acid solutions for 8 s under a microgravity environment realized in a drop shaft^7^. They concluded that gas bubble evolution under microgravity is strongly influenced by the wettability of the electrode in contact with the electrolyte. Sepahvandi et al. analyzed the structural, physicochemical, and thermodynamic parameters that govern hydrogel functionality to compare hydrogel performance in different environments: normal gravity and simulated microgravity^8^. They concluded that variables such as temperature gradient and osmotic pressure need to be controlled to resolve issues occurring under microgravity such as non-uniform crosslinking, anisotropic deformation, and instability during degradation. Furthermore, there are studies assessing how microgravity affects plants. Totsline et al. reported that lettuce is more susceptible to *Salmonella* infection under simulated microgravity than normal gravity^9^. Nakajima et al. found that simulated microgravity induces water uptake, resulting in enhanced seedling growth^10^. However, there have been no reported studies using spectroscopy to examine the dynamics of water under microgravity.

Near-infrared (NIR) spectroscopy is one of the most powerful tools for analyzing the HBN of liquid water^11–14^. In the NIR region, two absorption bands at approximately 1450 and 1920 nm can be assigned to ${\nu}_{1}+{\nu}_{3}$ and ${\nu}_{2}+{\nu}_{3}$ of water, respectively. In the infrared (IR) region, the bands at 2740 nm (ν_1_, O–H symmetric stretching), 6270 nm (ν_2_, H–O–H bending), and 2670 nm (ν_3_, O–H antisymmetric stretching) correspond to the fundamental vibrational modes of water^11–14^. The shift in the water absorption bands caused by changes in the HBN is much larger in the NIR region than in the IR region because the NIR region captures overtones and combinations of vibrations occurring in the IR region^11,14^. This sensitivity makes NIR spectroscopy particularly well suited to detecting variations in the HBN of water.

Factors such as temperature and ions are known to affect the HBN of water^15–19^. By harnessing the advantages of NIR spectroscopy mentioned above, many studies have investigated the dependence of the HBN of water on temperature and ionic interactions. For example, as the temperature increases, hydrogen bonds tend to break, resulting in a blue shift (i.e., a shift to shorter wavelengths) in the water absorption bands. This shift observed using vibrational spectroscopic methods, such as IR, Raman, and NIR spectroscopic methods, is often explained by modeling water as a mixture of different structural components^15–17,20–22^: weakly hydrogen bonded (WHB) water at the shorter wavelength and strongly hydrogen bonded (SHB) water at the longer wavelength. Šašić et al. concluded that more than 99% of water spectra can be explained in terms of only two water species: WHB and SHB water^15^. As the temperature increases, the contribution of WHB water increases while that of SHB water decreases, thus explaining the overall blue shift of the water band. Moreover, adding sodium salts of inorganic ions in the Hofmeister series^23,24^, which ranks ions on the basis of their salting-out strength, affects the water absorption bands. Strong salting-out salts cause a red shift (longer wavelengths), while weak salting-out ones induce a blue shift (shorter wavelengths), reflecting an expansion or disruption of the HBN, respectively^18,19^.

In this study, we used NIR spectroscopy to investigate whether the HBN of water molecules in ultrapure water and five types of ionic aqueous solutions changes under microgravity. A clinostat was used to create a simulated microgravity of less than 0.1 G by canceling gravity on a time-averaged basis. By acquiring NIR spectra under normal and microgravity, we evaluated the influence of gravity on the HBN of water. Furthermore, we investigated whether ion-induced changes in the HBN differ under low gravity.

These findings provide insight into how gravity influences the HBN of water. Even subtle gravity-induced changes in the HBN of water may disrupt finely balanced biochemical processes, possibly affecting human health. Understanding these effects is therefore essential for long-term space exploration as humanity moves toward sustained space habitation.

## Materials and methods

### Sample preparation

Ultrapure water (214–01301, FUJIFILM Pure Chemical Co., Japan) and five types of ionic aqueous solutions were analyzed using NIR spectroscopy. Five types of inorganic ions—Na_2_CO_3_, CH_3_COONa, NaCl, NaI, and NaSCN—were each diluted to a concentration of 1.0 mol/L with ultrapure water (214–01301).

These ions were selected because they share a common cation, Na⁺, and their salting-out strength follows the Hofmeister series. The Hofmeister series classifies ions on the basis of their ability to salt out or salt in proteins. According to this classification, the salting-out strength of the selected anions decreases as follows^23,24^: CO_3_^2−^ ˃ CH_3_COO^−^ ˃ Cl^−^ > I^−^ > SCN^−^. Among these ions, CO_3_^2−^ and CH_3_COO^−^ exhibit strong salting-out effects and are referred to as kosmotropic anions. These anions are known to enhance the HBN of water. In contrast, Cl^−^, I^−^, and SCN^−^ tend to salt in proteins and are classified as chaotropic anions, and they are reported to disrupt the HBN of water^18,25,26^.

### NIR spectroscopic measurements under clinostat-controlled gravity

**Fig. 1:**
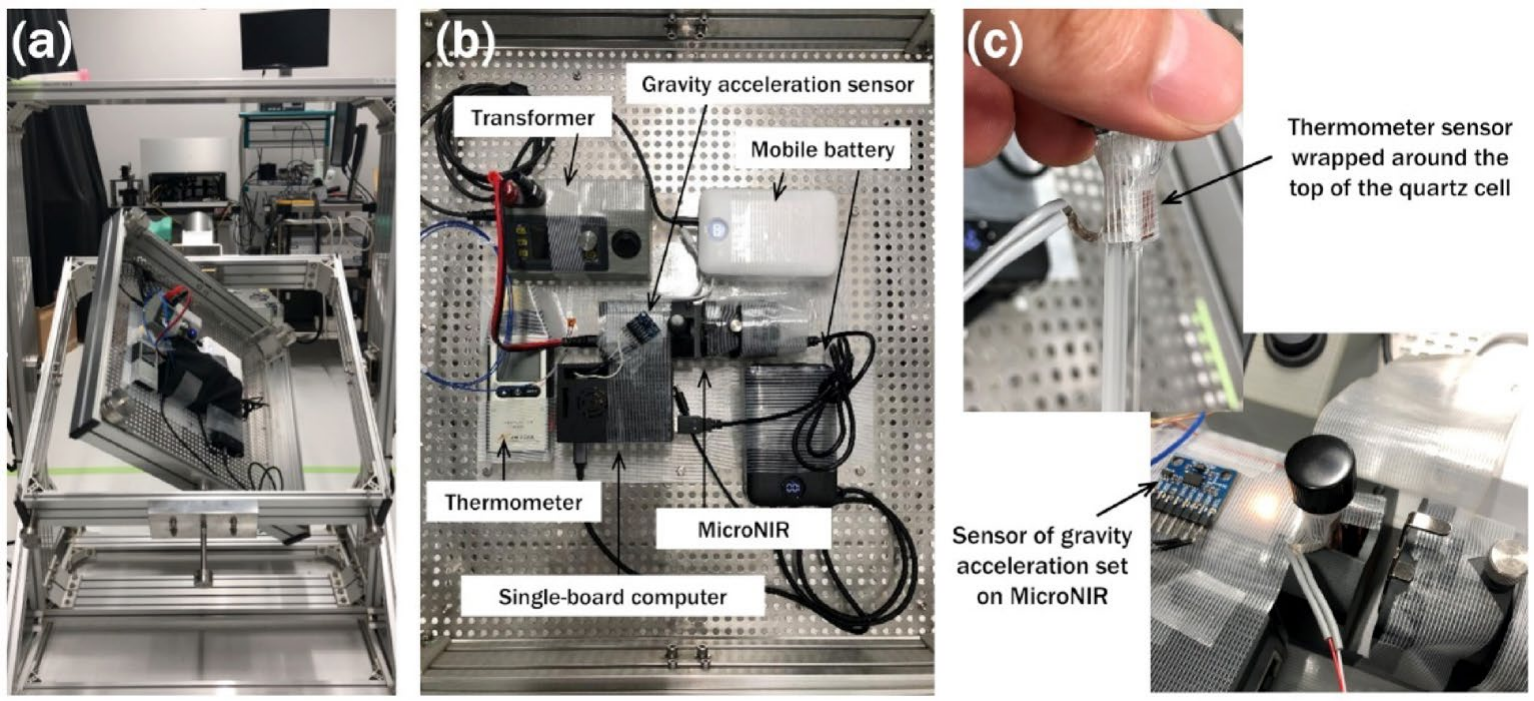
(**a**) NIR system on a clinostat with several devices to collect NIR spectra under simulated microgravity. (**b**) Detailed arrangement of the devices on the clinostat. (**c**) Setup of sensors of temperature and gravitational acceleration.

NIR spectra were recorded using a portable MicroNIR spectrometer (1700 ES, VIAVI Solutions Inc., USA). To enable NIR measurements under microgravity, the clinostat was equipped with a mobile battery (Anker Power Bank A1647, Anker Innovations Limited, China), transformer (DPS5005 DC-to-DC converter, Joy-it, Germany), temperature logger (TR-55i-TC, T&D Co., Japan), 3D gravitational acceleration sensor (GY-291, ADXL345, RobinEllis, Japan), and single-board computer (RaspberryPi4, Model B 8GB, Kyohritsu Electronic Industry Co., Ltd., Japan) for wireless data transfer to a laptop PC, in addition to the NIR spectrometer (Fig. 1a and b). NIR measurements were conducted in the transmission mode using a quartz cell with an optical path length of 1.0 mm (S15-IR-1, GL Sciences Inc., Japan).

The temperature was measured by wrapping a thermometer sensor around the top of the quartz cell (Fig. 1c). The temperature of water inside the cell was not directly measured; instead, the temperature at the surface of the quartz cell was recorded. A validation study was conducted to assess whether there was a significant difference between the surface temperature of the quartz cell and the temperature of water inside the cell. The method for verifying the accuracy of temperature monitoring in the NIR system mounted on the clinostat is provided in detail in Supporting Information (SI) 1. Approximately 10 min after the cell was placed in the NIR spectroscopic system, the temperature readings at both the cell surface and cell interior were found to be nearly identical in both static and rotating states (Figure S1). Therefore, NIR measurements started at least 10 min after cell placement to ensure thermal equilibrium. Under these conditions, there was no meaningful temperature difference between the cell surface and water inside the cell, and thus, here, the term “temperature” referred to both.

Although the NIR spectrum of water was affected by temperature, the NIR system used in this study did not include a temperature controller. Without active temperature control, it was impossible to isolate the effects of gravity on the HBN of water. To address this issue, an analysis method was developed by limiting the temperature range of the NIR spectra analyzed—such as performing PCA on datasets without significant temperature differences between static and rotating conditions. The procedure for determining the optimal conditions for data analysis without active temperature control is described in detail in Results and Discussion.

The NIR spectra of ultrapure water with temperature variations were collected as reference data using an IRTracer-100 NIR system (Shimadzu, Japan), open quartz cell with an optical path length of 0.1 mm (AB20-IR-0.1, GL Sciences Inc., Japan), and digital temperature controller (PN 076-1510, Pike Technologies, USA) under a flow of dry air (Precision Nitrogen Trace 1000, PEAK Scientific, UK).

### Gravity control with a 3D clinostat

To establish a microgravity environment, the sample was rotated around two axes using a 3D clinostat, which cancelled gravity over time by averaging the gravitational vector. The rotation speed was set to 6 revolutions per minute (rpm) along both axes. Figure S2 in SI 2 shows an example of measuring the gravitational acceleration acting on a sample placed on the clinostat using a gravitational acceleration sensor (Fig. 1). In addition, the effects of rotation on the NIR instrument were also investigated (Figure S3 in SI 2). The detailed discussion for the basic experimental conditions is provided in SI 2.

### Spectral data analysis

NIR spectra with 6.2 nm resolution were preprocessed by interpolation with intervals of 2 nm and baseline correction. Principal component analysis (PCA)^27^ was conducted on preprocessed NIR spectral data using a chemometrics software, Unscrambler X 10.3 (Camo Analytics, Oslo, Norway), to examine variations in the HBN of water.

To determine whether the obtained PCs were correlated with temperature or rotation (presence or absence), correlation coefficients between the PCA scores and each of these variables were calculated. A binary variable representing the presence or absence of rotation was defined and referred to as the gravitational function, where a value of 0 was assigned to the static state and 1 to the rotating state.

Student’s *t*-test was adopted to evaluate statistically significant differences between the mean values of the two groups. A threshold *p*-value of 0.05 was established to determine the existence of significant differences.

## Results

### Variations in HBN of ultrapure water with temperature

**Fig. 2:**
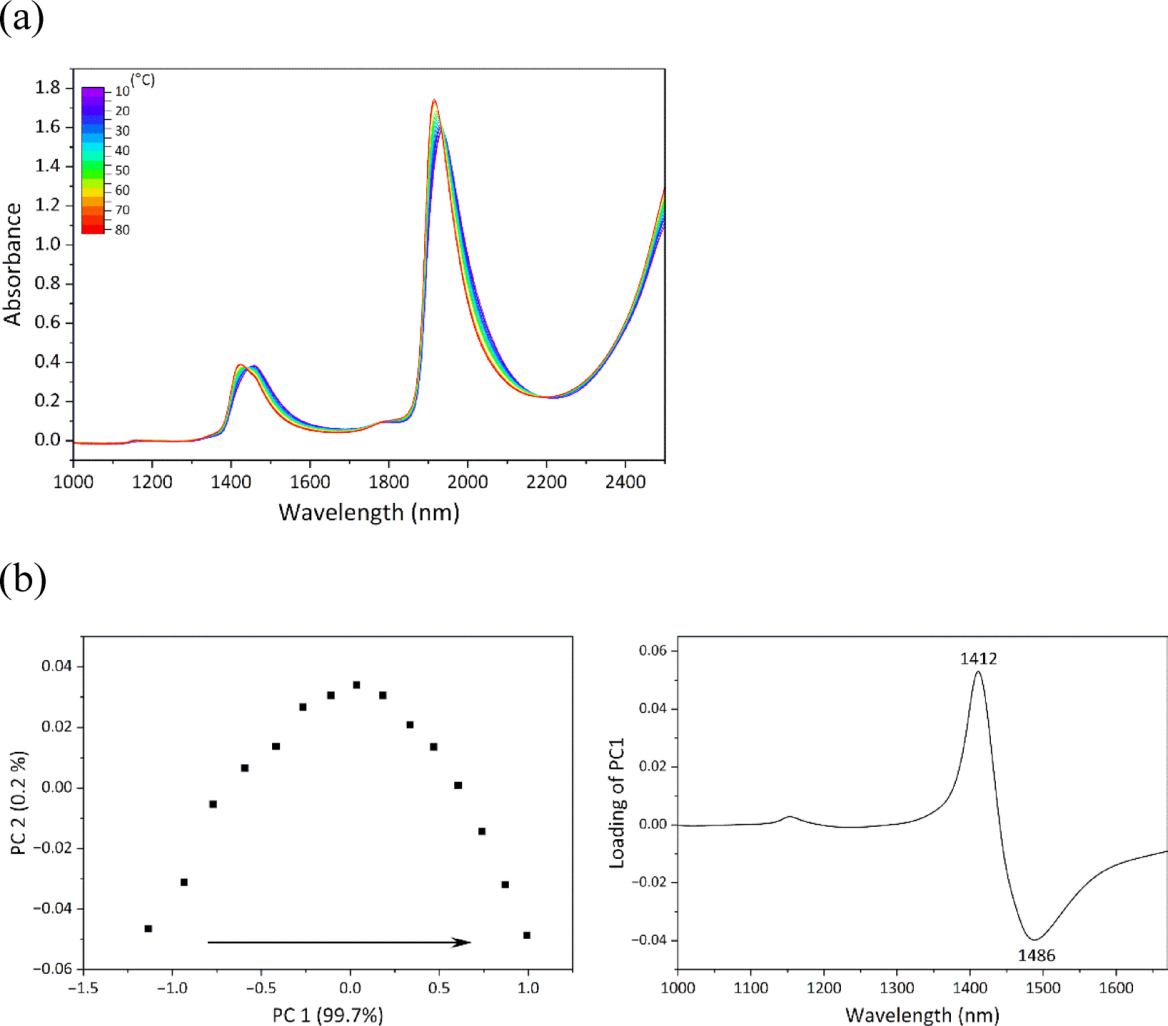
(**a**) NIR spectra of ultrapure water in the region of 1000–2500 nm acquired by the benchtop NIR system in the temperature range of 10–80 °C at intervals of 5 °C. (**b**) Score (PC1 vs. PC2) and loading (PC1) plots of PCA conducted on NIR spectral data of ultrapure water in the region of 1000–1670 nm with temperature variations of 10–80 °C.

To investigate changes in the HBN of water molecules under different gravitational conditions, we first examined in detail how the NIR spectrum of water responded to HBN changes induced by temperature variations. Figure 2a shows NIR spectra in the region of 1000–2500 nm obtained by the benchtop NIR system at temperatures of 10–80 °C with intervals of 5 °C. Increasing the temperature shifted the water bands near 1450 and 1920 nm to shorter wavelengths. As the temperature increased, hydrogen bonds between water molecules tended to break, and the water absorption bands exhibited a blue shift corresponding to changes in the HBN^15–17,20^.

To analyze changes in the absorption bands of water with temperature variation, PCA was performed by focusing on the region of 1000–1670 nm, which contained overlapping peaks from spectra obtained by the benchtop NIR system and the portable NIR system. The PCA score plot (PC1 vs. PC2) and PC1 loading plot are shown in Fig. 2b. With increasing temperature, the PC1 score increased monotonically, indicating that the spectral component represented by PC1 loading contributed more strongly to the water spectra at higher temperatures. In the PC1 loading plot, a positive peak was observed at 1412 nm and a negative peak at 1486 nm. These features indicate an overall shift of the water band toward shorter wavelengths with increasing temperature. Thus, band shifts associated with changes in the HBN of water can be systematically analyzed using PCA. A detailed discussion for the band shifts represented by PCA is provided in the Supporting Information (SI 3, Figure S4).

### NIR spectra of ultrapure water collected by the portable NIR spectroscopic system mounted on the clinostat

**Fig. 3:**
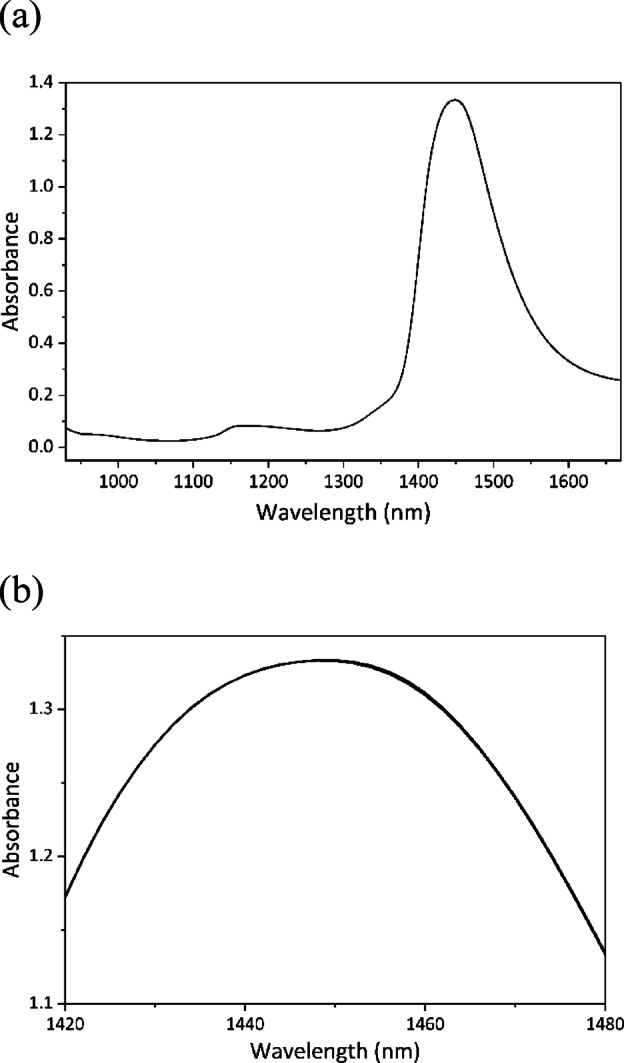
(**a**) NIR spectra of ultrapure water in the region of 930–1670 nm obtained by the portable NIR system mounted on the clinostat every 5 min for 40 min under static conditions. Measurements started 20 min after setting the sample in the NIR system. (**b**) Enlarged view of the spectra in the region of 1420–1480 nm.

To accurately interpret the characteristics of the NIR spectra acquired using the NIR spectroscopic system without active temperature control mounted on the clinostat, the thermal stability of the NIR system and associated spectral variations were evaluated under static and rotating conditions. NIR spectra were acquired three times in both static and rotating modes to investigate the relationship between the thermal stability of the system and the resulting NIR spectra. Figure 3a presents the NIR spectrum of ultrapure water in the region of 930–1670 nm recorded in the static mode, and Fig. 3b is an enlarged view of the spectrum in the region of 1420–1480 nm. The band at 1450 nm is attributed to the combination of symmetric and antisymmetric O–H stretching modes (${\nu}_{1}+{\nu}_{3}$)^20,28,29^. Spectra were collected 20 min after the cell was settled into the system at intervals of 5 min over a period of 40 min. The average temperature was 29.9 °C, with fluctuations kept within ± 0.1 °C (named 1st in Figure S5a). As a result, the nine spectra mostly overlapped, exhibiting minimal variations (Fig. 3b). Repeating the experiment under the same conditions reconfirmed that temperature fluctuations could be suppressed to less than ± 0.1 °C of the average temperature (named 2nd in Figure S5a).

Larger temperature variations were intentionally introduced by conducting the measurements in the early morning—when the instrument temperature was low—even after allowing the instrument to stabilize for 1 h in the third trial (named 3rd in Figure S5a). Among the three trials, this condition induced the largest temperature fluctuation, reaching ± 0.9 °C. Figure S5b shows an enlarged view of the spectrum in the region of 1420–1480 nm obtained during the third trial. A noticeable blue shift in the water absorption band was observed with increasing temperature. These spectral variations were consistent with the variations in the temperature-dependent NIR spectra (Fig. 2). This confirmed that the system was capable of acquiring reliable NIR spectra under moderate thermal control by monitoring the water temperature, even in the absence of active temperature regulation.

Next, three samples of ultrapure water previously measured every 5 min under static conditions were subjected to rotation using the clinostat, and NIR spectra were continuously acquired. NIR measurements began 20 min after the start of clinostat rotation, at which point the effective gravity acting on the sample had decreased to less than 0.1 G (Figure S2). Rotating the clinostat slightly cooled the system, lowering the mean temperature by approximately 1 °C (Figures S1a, S1c, and S5c). However, temperature fluctuations during 40 min of measurement remained within ± 0.5 °C, confirming once again that stable NIR spectra could be obtained under rotational conditions, even without active temperature control.

### Effect of gravity on HBN of water from detailed NIR analysis

As described in the previous subsection, stable acquisition of NIR spectra was achieved under both static and rotating conditions while monitoring the temperature, despite the lack of precise temperature control. To statistically investigate changes in the HBN of water induced by gravity changes—independent of temperature variations—additional NIR data were collected under both static and rotating conditions. For each measurement series, NIR spectra were collected every 5 min for 60 min after 10 min of stabilization following installation of the quartz cell into the system. This was followed by activation of the clinostat, after which spectra were continuously acquired every 5 min for another 60 min. This procedure was repeated 10 times. In addition, 60 min of measurements under static and rotating conditions were independently repeated 7 and 14 times, respectively. Temperature variations during these measurements are shown in Figure S6a, with fluctuations of up to 5 °C depending on the measurement day and operational conditions of the NIR system.

**Fig. 4:**
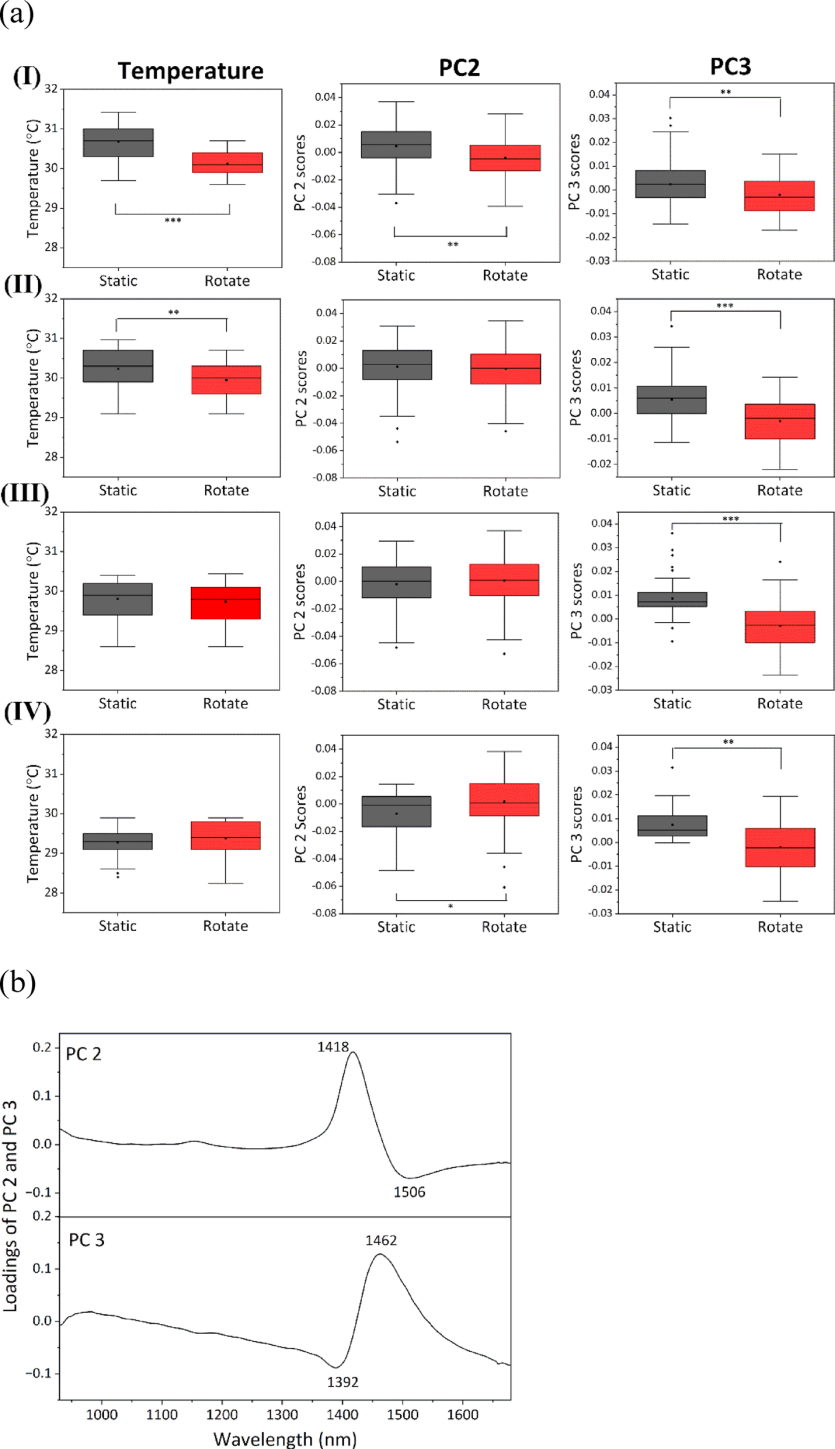
(**a**) Box plots of temperature and scores of PC2 and PC3 corresponding to temperature ranges I‒IV. (**b**) Loading plots of PC2 and PC3. (* *p* < 10^− 2^, ** *p* < 10^− 4^, *** *p* < < 10^− 6^)

PCA of all spectral data, obtained under both static and rotating conditions, was conducted to investigate whether differences in the HBN of water could be attributed to the gravity change. Figure S6b shows the score plots of PC3 and PC5, with PC3 exhibiting the strongest tendency to separate the data into static and rotating groups. However, the separation was not distinct, and no definitive effect of gravity could be concluded. One possible reason for the unsatisfactory PCA result is the variation in the NIR spectra caused by temperature fluctuations of up to 5 °C (Figure S6a). In the NIR system used in this study, precise temperature control was not available; therefore, the acquired spectra were influenced by both temperature and gravity. In the measurement series combining static and rotating modes, a temperature decrease was typically observed after the onset of rotation. Figure S6c indicates that the temperature was significantly lower in the rotating state than in the static state. To isolate the effect of gravity on the HBN of water, PCA was repeated using temperature-constrained data. The analysis was conducted within a temperature range of 2.0 °C, shifting the window by decrements of 0.5 °C. The temperature ranges applied are summarized in Table S1 of SI 4. Box plots showing temperature distributions for temperature ranges I–IV (Fig. 4a) were prepared to assess potential temperature bias between static and rotating states. In the higher temperature ranges (I and II), significant differences in the temperature were observed between the two conditions. In contrast, no such differences were found in the lower temperature ranges (III and IV). This trend was attributed to the imbalance in sample distribution, with static measurements more frequently associated with higher temperatures, and rotational measurements with lower temperatures—confirming again the tendency of temperature to slightly decrease upon rotation.

Subsequent PCA of NIR spectral data picked up PCA scores corresponding to each of temperature ranges I‒IV. Table S2 summarizes the *p*-values between static and rotating states calculated for the PCA scores for all temperature ranges. In all temperature ranges, significant differences were observed in PC3 and PC5. The score plots for PC3 and PC5 are shown in Figure S7, providing a clearer separation between the two groups compared with Figure S6b. In contrast, significant differences in PC2 were observed in some temperature ranges (Table S2). PC5 provided important information about the spectral variation caused by gravity. However, the contribution to the spectral variation from the higher order PC was small (< 0.1%). Therefore, changes in the water bands is primarily discussed on the basis of PC2 (13.5%−14.0%) and PC3 (4.6%−8.2%).

PC2 and PC3 scores for the highest temperature range, I (29.5–31.5 °C), were significantly different between static and rotating states (Fig. 4a). The loading plots of PC2 and PC3 are depicted in Fig. 4b. In the loading plot of PC2, the positive peak was observed at 1418 nm and the negative peak at 1506 nm. In the loading plot of PC 3, the negative peak was extracted at 1392 nm and the positive peak at 1462 nm. In temperature range I, PC2 and PC3 scores were statistically smaller in the rotating state than in the static state. Therefore, PC2 and PC3 acted as components that increased the contributions of SHB and WHB water in the rotating state, respectively. Because PC2 had a higher contribution to total spectral variations than PC3, the band intensity at the longer wavelength attributed to SHB water was stronger in the rotating state than in the static state. Therefore, the result indicated that the HBN of water was stronger under microgravity than under natural gravity in temperature range I.

PCA of data obtained in temperature ranges II‒IV was similarly conducted, and the resulting loadings were consistent with those in temperature range I, as shown in Fig. 4b. However, the distribution of PC2 scores varied depending on the temperature. As shown in Fig. 4a, in temperature range II, PC2 scores tended to be higher in the static state than in the rotating state, similar to the trend observed in temperature range I. In contrast, the opposite trend was observed in temperature ranges III and IV, with higher PC2 scores in the rotating state than in the static state. Interestingly, PC3 scores were consistently and significantly higher in the static state than in the rotating state across all temperature ranges (I–IV), regardless of whether a temperature difference existed between the static and rotating states. This suggests that PC3 captures spectral components associated with changes in the HBN induced by variations in gravitational conditions. Moreover, the observed PC3 values indicated that the band intensities at 1460 and 1400 nm were decreased and increased, respectively, under microgravity; namely, the band tended to shift toward shorter wavelength. Therefore, the HBN of water tends to weaken under microgravity.

In temperature regions I and II, strengthening of the HBN of water as the gravity decreased from normal gravity to microgravity can be attributed to two main factors: (1) The temperature is statistically lower in the rotating state than in the static state and (2) NIR spectral characteristics are less affected by gravity-induced HBN variations than residual temperature-related variations, even within the restricted 2 °C range. To accurately evaluate the effects of gravity on the HBN of water, it is at least necessary to analyze the dataset within a narrow temperature range (e.g., 2 °C), ensuring that there is no significant temperature difference between static and rotating conditions.

### Effect of ions on the HBN of water

**Fig. 5:**
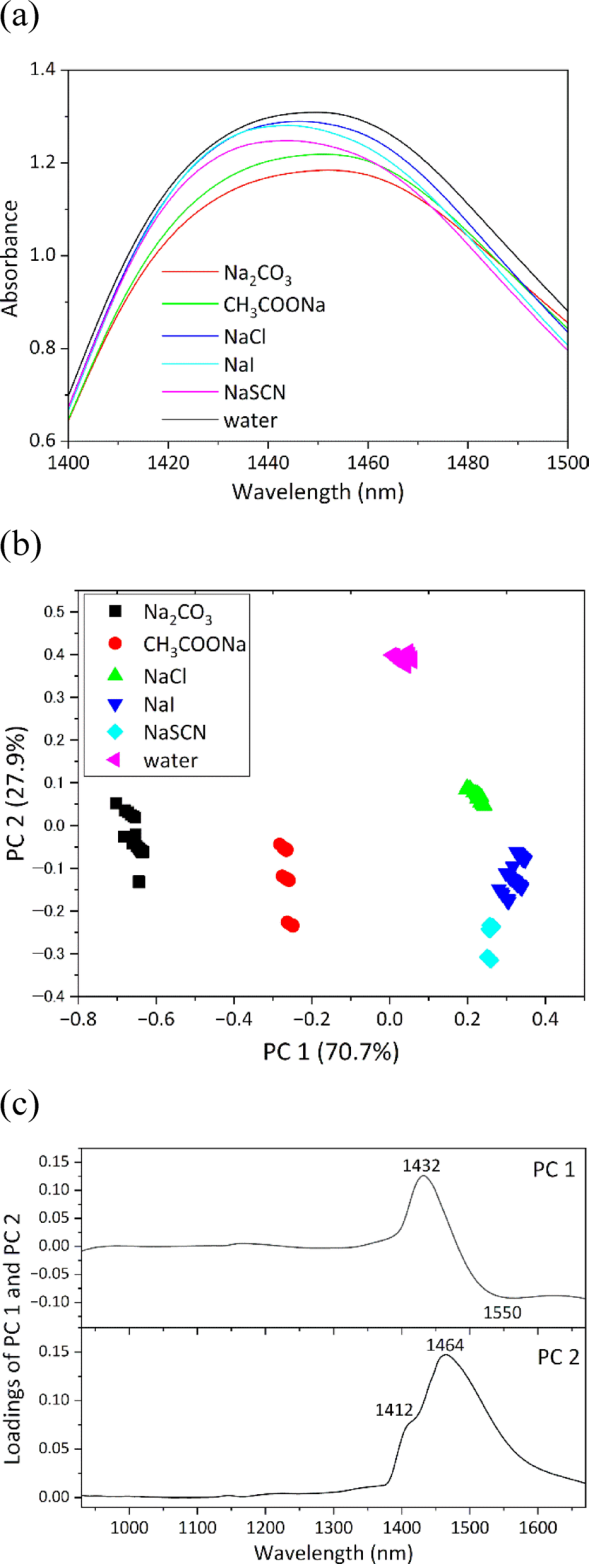
(**a**) Mean NIR spectra of 1.0 mol/L ionic aqueous solutions and ultrapure water in the region of 1400–1500 nm obtained under static conditions at 28.0 ± 0.5 °C. (**b**) Score plots and (**c**) loading plots of PCA conducted on dataset of five types of ionic aqueous solutions and ultrapure obtained under static conditions in the range of 27.0–30.0 °C.

Five types of inorganic salts (CO_3_^2−^, CH_3_COO⁻, Cl⁻, I⁻, and SCN⁻) were selected on the basis of their position in the Hofmeister series, all containing Na^+^ as the common cation. These anions induce salting-out effects in the order of CO_3_^2−^ > CH_3_COO⁻ > Cl⁻ > I⁻ > SCN⁻. Figure 5a shows the mean NIR spectra of 1.0 mol/L aqueous solutions of each salt, as well as ultrapure water, in the region of 1400–1500 nm. The mean spectra were calculated from data measured at 28.0 ± 0.5 °C under static conditions. Because CO_3_^2^⁻ and CH_3_COO⁻ induced strong salting-out effects, they also shifted the absorption bands of water to longer wavelengths compared with those of pure water. In contrast, because Cl⁻, I⁻, and SCN⁻ induced weaker salting-out effects, they shifted these bands to shorter wavelengths compared with those of pure water. Moreover, the degree of these spectral shifts followed the exact order of the Hofmeister series: CO_3_^2−^ > CH_3_COO^−^ > Cl^−^ > I^−^ > SCN^−^. This trend is consistent with that observed in previous studies^18,30–32^, demonstrating that CO_3_^2−^ and CH_3_COO⁻, as kosmotropic anions, strengthen the HBN of water, whereas Cl⁻, I⁻, and SCN⁻, as chaotropic anions, disrupt or weaken the HBN of water.

To systematically investigate the differences in the HBN of water resulting from salt dissolution, PCA was conducted on the NIR spectral datasets of these five ions in aqueous solutions and ultrapure water, which were measured under static conditions in the temperature range of 27.0–30.0 °C. In PC1, the ions were ordered from positive to negative scores in accordance with their salting-out strength (Fig. 5b). The plots of ultrapure water were located between those of CH_3_COONa and NaCl, which was consistent with the direction of spectral band shifts relative to the water band shown in Fig. 5a. The PC1 loading plot exhibited a positive peak at 1432 nm and a negative peak at 1550 nm, indicating that water band shifted toward longer wavelength in the presence of kosmotropic anions compared with chaotropic anions, in agreement with the Hofmeister series. Ultrapure water and salt solutions were separated along PC2 (Fig. 5c). PC2 loading plots showed positive peaks spanning the broad wavelength region of the water band, suggesting that PC2 primarily reflected spectral changes caused by a decrease in the water content with salt addition. These results clarify that PC1 primarily captures changes in the HBN of water induced by ion addition, which depended on the anion’s order in the Hofmeister series.

### Effect of gravity on the HBN of water in ionic solutions

**Fig. 6:**
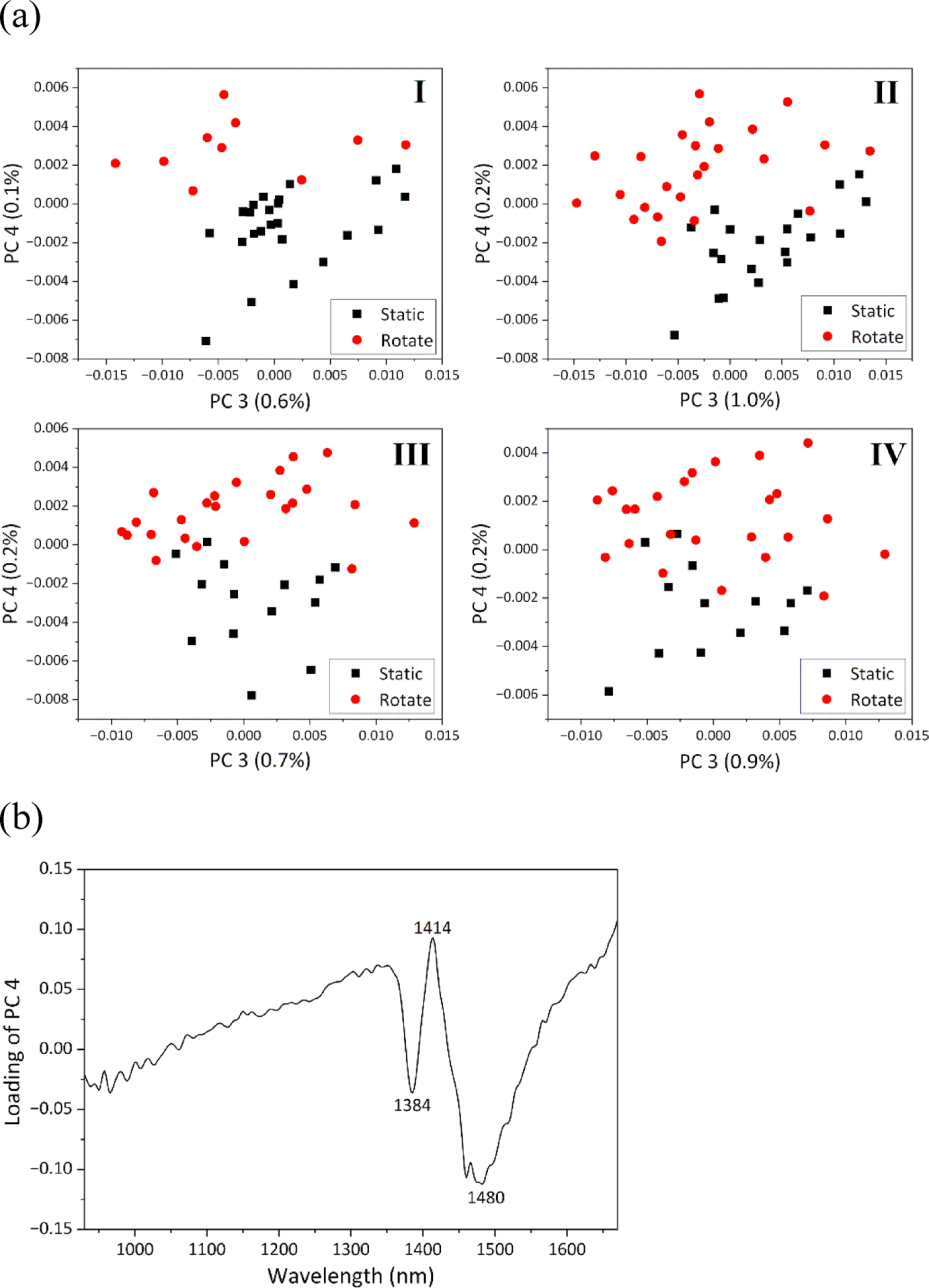
(**a**) Score plots of PC3 vs. PC4 in all temperature ranges. (**b**) Loading plot of PC4 in temperature range III.

In the next step, the effect of gravity on the HBN of water containing ions was investigated. Here, we discuss in detail the CH_3_COONa aqueous solution as a representative of the five ionic aqueous solutions studied. PCA was conducted on datasets divided into intervals of 2 °C, similar to the approach used in the analysis of ultrapure water. Definitions of the temperature ranges and corresponding *p*-values for the temperature differences between static and rotating states are summarized in Table S3. Representative score plots (PC3 vs. PC4) in all temperature ranges and the loading plot of PC4 in temperature range III are shown in Fig. 6a and b, respectively. In each temperature range, the data were separated into two groups—static and rotating—on the basis of PC4 scores (Fig. 6a). The correlation between PC4 scores and gravitational function exceeded 0.69 across all temperature ranges (Table S3), indicating that PC4 captured spectral changes caused by gravity-induced HBN changes. The direction of the PC4 score bias (positive) and loading peaks (positive loading at 1414 nm and negative loadings at 1384 nm and 1480 nm) in the rotating state (Fig. 6a and b) indicated that the HBN weakened under microgravity in all temperature ranges.

Similarly, PCA was independently performed on the dataset of each ionic solution, revealing no significant temperature differences between static and rotating states. The analysis confirmed that the HBN weakened as the gravity decreased from normal gravity to microgravity across all ionic solutions—consistent with the behavior previously observed in ultrapure water. PCA results for each solution are shown in Figure S8.

### Effect of salting-out strength on gravity-induced alterations in HBN of water

**Fig. 7:**
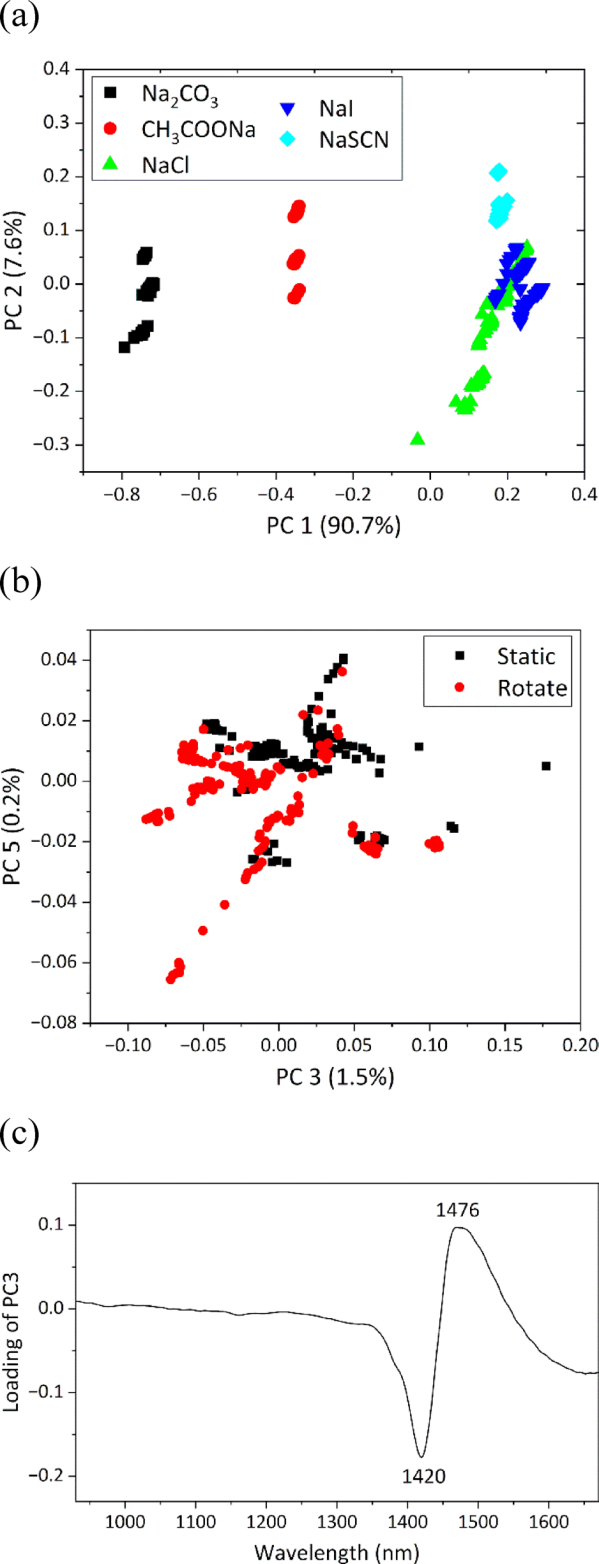
Score plots of (**a**) PC1 vs. PC2 and (**b**) PC3 vs. PC 5 and (**c**) PC3 loading plots of PCA conducted on dataset of five types of ionic aqueous solutions obtained under static and rotating conditions.

To examine the effect of gravity on the HBN of ion-containing water across the Hofmeister series, PCA was conducted on the combined NIR spectral dataset of the five types of ionic aqueous solutions under static and rotating conditions. The PC1 score plot in Fig. 7a displayed a distribution pattern consistent with the Hofmeister series, similar to that observed in Fig. 5b. PC1 captured the spectral variance caused by different ionic species, indicating that ionic effects on the HBN were larger than gravitational effects. Next, PCs associated with gravity-induced changes in the HBN of water were investigated. PC3 and PC5 were found to slightly separate the data into two distinct groups corresponding to static and rotating conditions (Fig. 7b). In the PC3 loading plot, a negative peak was observed at 1420 nm and a positive peak at 1476 nm (Fig. 7c), and PC3 scores were biased toward negative values under the rotating condition (Fig. 7b). These results suggest that PC3 represents the spectral features associated with weakening of the HBN of water under microgravity. This trend was consistent with the findings from analyzing an individual ionic solution (Figs. 6 and S8). However, PCA of the dataset of each ionic solution showed stronger correlations with the gravitational function, with correlation coefficients exceeding 0.62 (Table S4). Therefore, the correlation between PC3 scores and the gravitational function was recalculated for each ion. As a result, PC3 and the gravitational function scores showed little to no correlation in solutions containing kosmotropic anions (Na_2_CO_3_ and CH_3_COONa) but very strong correlation in solutions containing chaotropic anions (NaCl, NaI, and NaSCN) (Table S5).

**Fig. 8:**
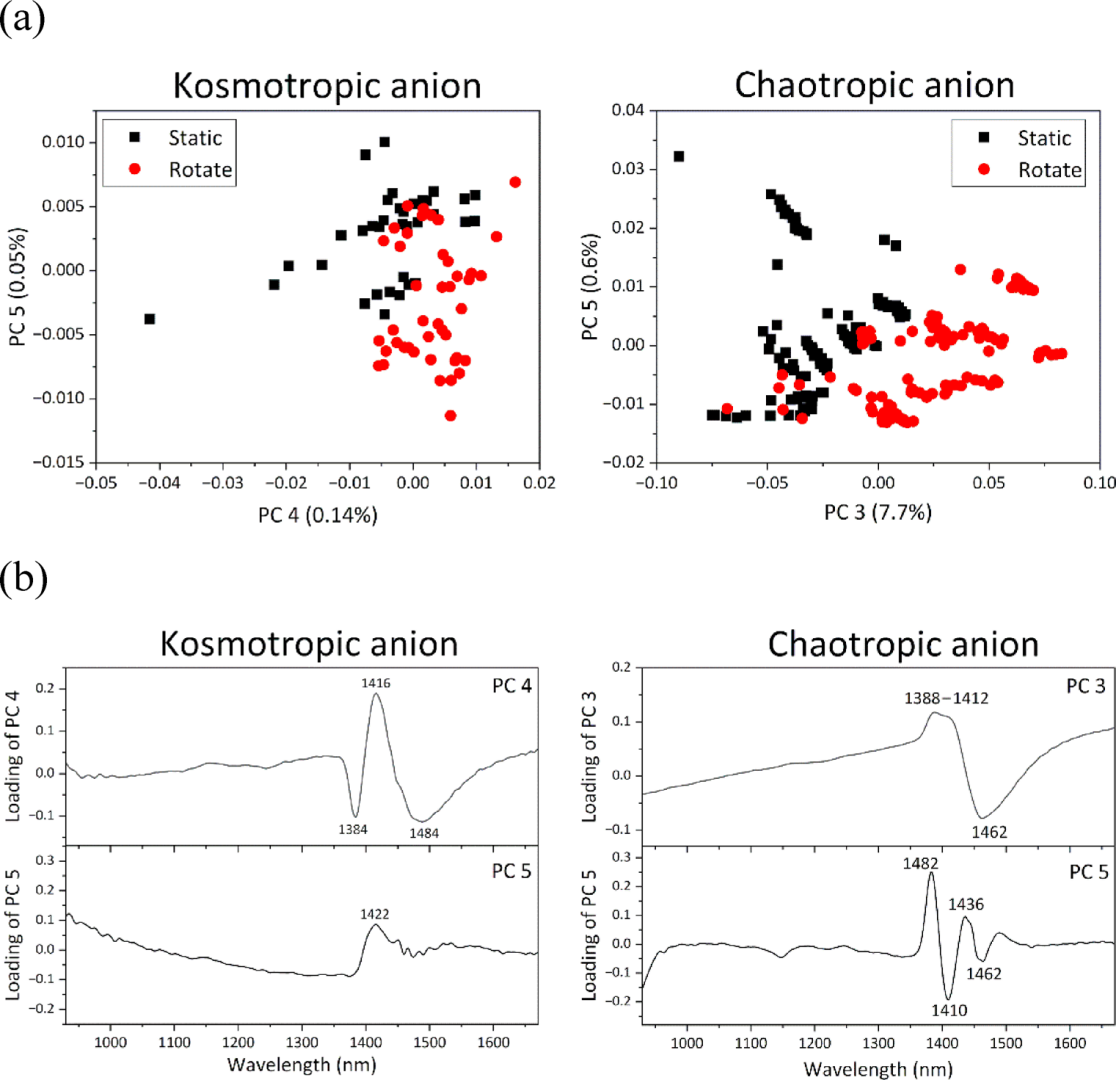
(**a**) Score and (**b**) loading plots of PCA conducted on datasets of kosmotropic anions (Na_2_CO_3_ and CH_3_COONa, PC4 vs. PC5) and chaotropic anions (NaCl, NaI, and NaSCN, PC3 vs. PC5).

PCA was separately conducted on the datasets of kosmotropic and chaotropic anions. Data of kosmotropic anions were classified into two groups (static and rotating states) by PC4 and PC5, whereas data of chaotropic anions were grouped by PC3 and PC5 (Fig. 8a). Both PC4 for kosmotropic ions and PC3 values for chaotropic anions were biased toward positive values (Fig. 8a). Moreover, their corresponding loadings exhibited positive peaks at 1416 nm and the range of 1388–1412 nm (Fig. 8b), indicating that the water absorption band blue-shifted under microgravity. When PCA was conducted on the five types of ionic solutions together, weakening of the HBN of water under microgravity observed in the presence of chaotropic anions was not clearly detected in the presence of kosmotoropic anions, as explained above. Therefore, changes in the HBN of water were likely smaller in the presence of kosmotoropic anions than in the presence of chaotropic anions. Consequently, it was difficult to detect slight weakening of the HBN of water under microgravity in the presence of kosmotoropic anions because the HBN of water was strengthened by adding kosmotoropic anions. In contrast, weakening of the HBN of water under microgravity in the presence of chaotropic anions was easily detected because the HBN of water was already weakened by adding these anions, and water molecules predominantly existed in a free state unbound to each other. It is particularly interesting that the different effects of these ions on the HBN of water, arising from their different salting-out strengths, are reflected in the different dynamics of water molecules under microgravity, namely the tendency toward the free or bound state.

## Discussion

In this study, we evaluated the effect of gravity on the HBN of water using NIR spectroscopy. The HBN between water molecules in both ultrapure water and five types of ionic solutions consistently weakened under microgravity after excluding the influence of temperature. Moreover, gravitational changes had a smaller effect on the HBN of ultrapure water than temperature changes of even a few degrees. In addition, gravity-induced weakening of the HBN was less apparent in solutions containing kosmotropic anions than in solutions containing chaotropic anions.

Here, we discuss why the HBN in water tends to weaken under microgravity. To date, there have been very few investigations into the intrinsic dynamics of water molecules under microgravity, and as mentioned in the Introduction, no spectroscopic studies have been reported that directly examine water dynamics under such conditions. Nevertheless, insights can be gained by drawing analogies from previous studies that have explored the effects of pressure- and density-induced perturbations on water. Huš et al. evaluated the dependence of hydrogen bond strength on local molecular environments—such as molecular arrangement, coordination number, and intermolecular distance—using quantum mechanical methods^33^. They demonstrated a negative correlation between hydrogen bond strength and intermolecular distance: hydrogen bonds become stronger as the distance between water molecules decreases, whereas increasing intermolecular separation leads to weaker hydrogen bonding. Furthermore, Song et al. employed a density functional theory–based deep neural network approach to investigate the dielectric properties of liquid water under pressures ranging from 0.1 MPa to 1000 MPa^34^. Under high-pressure conditions, compression increases water density and shortens the O$\cdots$O distances, resulting in an enhanced molecular dipole moment and strengthened hydrogen bonding. Consistently, Noguchi et al. reported near-infrared spectra of H_2_O measured under high-pressure and high-temperature conditions (up to 368 °C and 16 GPa)^35^. They observed that the combination (ν₂ + ν₃) and overtone (2ν₃) bands in the regions of 4500–5500 cm⁻¹ and 6000–7000 cm⁻¹, respectively, shifted toward lower wavenumbers with increasing pressure, suggesting a redistribution toward more extensively hydrogen-bonded H_2_O clusters.

Collectively, these studies consistently indicate that hydrogen bonding in water is strengthened under high-pressure conditions. In contrast, there are very few reports that quantitatively examine liquid water—either experimentally or through simulations—under reduced-pressure conditions. However, it is reasonable to expect that water dynamics under low pressure exhibit trends opposite to those observed at high pressure. Specifically, reduced pressure is anticipated to induce an expansion of the O$\cdots$O intermolecular distances and a decrease in water density, both of which would lead to a weakening of the HBN.

The behavior of water under microgravity can further be inferred from its pressure-dependent dynamics. JAXA has reported that, in a microgravity environment, hydrostatic pressure is effectively absent for both liquids and gases, owing to the lack of significant fluid weight to generate a pressure gradient^36^. Under normal gravity, hydrostatic pressure increases with depth in a fluid column, leading to a pressure gradient between the upper and lower regions. Because an increase in hydrostatic pressure corresponds to an increase in internal pressure, intermolecular distances tend to shift toward compression^37,38^. However, because liquid water is nearly incompressible, these changes are extremely small: even in Earth’s oceans, where depths of several thousand meters correspond to hydrostatic pressures on the order of 100–400 atm or higher, the resulting density increase is only a few percent at most^39,40^. In microgravity, the effective cancellation of fluid weight eliminates the hydrostatic pressure gradient. Consequently, phenomena associated with hydrostatic compression do not occur, and intermolecular distances may instead shift slightly in the opposite direction, namely toward expansion. From this perspective, the observed tendency for the HBN in water to weaken under microgravity conditions is a plausible and physically consistent outcome. Moreover, it is noteworthy that the effects of salts on the hydrogen bond network are consistently observed as systematic trends across all five ionic aqueous solutions, without contradiction, within the overall framework of HBN weakening under microgravity.

Living organisms contain various ions, such as bicarbonate (HCO_3_⁻) and phosphate (HPO_4_^2^⁻), which act as buffers and play key roles in maintaining physiological functions. Although the HBN of water was weakened under microgravity in the presence of ions, the degree of weakening varied depending on the ion species, reflecting their different effects on the HBN. These findings suggest that even slight changes in the HBN caused by changes in gravity may disrupt delicately balanced biochemical reactions, potentially impacting human health.

As humans prepare for life in space, understanding the effects of gravity on biological systems is essential. Because the human body is mostly composed of water, it is crucial to first understand the dynamics of water molecules under different gravitational conditions through fundamental research. We hope this study will serve as a foundation for future investigations into the relationship between water molecular dynamics, human health, and the origin of life in space.

## Supplementary Information

Below is the link to the electronic supplementary material.

Supplementary Material 1

## Data Availability

The data that support the findings of this study are available from the corresponding author upon reasonable request.
