# Supplementary material for: Gravitational effects on the hydrogen bond network of water and ionic solutions revealed by near infrared spectroscopy under simulated microgravity
Source: Sci Rep. 2026 Mar 14;16:13497. doi: 10.1038/s41598-026-44169-1 (PMC13111628; doi:10.1038/s41598-026-44169-1)
Supplement: Supplementary file 1 — Supplementary Material 1 [file 41598_2026_44169_MOESM1_ESM.pdf]

[Supporting Information]

Gravitational Effects on the Hydrogen Bond Network of Water and Ionic Solutions Revealed by Near Infrared Spectroscopy under Simulated Microgravity

Mika Ishigaki[1]*, Koyo Koizumi[2], Kotomi Asano[1], Naoki Okamoto[2], Go Takehi[2], Riku Sasamoto[3], Masato Takeuchi[3], Roumiana Tsenkova[4], Mio Matsui[5], Aiko Nagamatsu[5], Mariko Egawa[2]*

[1] *Institute of Agricultural and Life Sciences, Academic Assembly, Shimane University, Japan*
[2] *MIRAI Technology Institute, Shiseido Co., Ltd., Japan*
[3] *Department of Applied Chemistry, Graduate School of Engineering, Osaka Metropolitan University, Japan*
[4] *Graduate School of Agricultural Science, Kobe University, Japan*
[5] *Space Exploration Innovation Hub Center, Japan Aerospace Exploration Agency*

*Authors to whom correspondence should be sent.
*E-mail: ishigaki@life.shimane-u.ac.jp (M.I.)
mariko.egawa@shiseido.com (M.E.)

SI 1. Verification of the accuracy of temperature monitoring in a NIR system on the clinostat
SI 2. Verification of the microgravity environment established by the 3D clinostat
SI 3. Detailed discussion of the temperature-dependent NIR band shifts using PCA
SI 4. Supporting figures, table, and statistical analysis for ultrapure water
SI 5. Supporting figures, table, and statistical analysis for ion solutions

SI 1. Verification of the accuracy of temperature monitoring in a NIR system on the clinostat

The NIR system does not have a built-in temperature control module. Because the NIR spectrum of water is highly sensitive to temperature, accurately monitoring the water temperature is essential to analyze the characteristics of the acquired spectra. Ideally, the temperature of water inside the cell should be recorded by inserting a temperature sensor directly into the cell through the cap. However, owing to water spillage as the clinostat rotated, this setup was not feasible. Instead, the temperature sensor was wrapped around the top of the quartz cell to measure the temperature (Figure 1c). Therefore, this method recorded the temperature at the surface of the quartz cell rather than the temperature of water inside the cell. Therefore, it was necessary to evaluate whether the temperature at the cell surface was significantly different from the temperature of water inside the cell. Figure S1a shows the temporal variations in the water temperature (T_{water}) and cell surface temperature (T_{cell}) after the cell was placed in the NIR system under static conditions, while Figure S1b presents the temperature difference ($T_{\mathrm{cell}} - T_{\mathrm{water}}$). Data were similarly obtained under rotating conditions (Figure S1c and S1d). The results confirmed that the temperature stabilized approximately 10 min after cell placement, and the temperature difference remained within 0.41 ± 0.08 °C under static conditions and within 0.62 ± 0.06 °C under rotating conditions (Figure S1b and S1d). Additionally, both the water and cell surface temperatures under rotating conditions were approximately 1.0 °C lower than those under static conditions (Figure S1b and S1d). This temperature decrease was attributed to an enhancement in heat dissipation through clinostat rotation. However, because this temperature drop occurred equally at both the cell surface and cell interior, the temperature difference ($T_{\mathrm{cell}} - T_{\mathrm{water}}$) was unaffected by clinostat rotation. Therefore, to ensure temperature stability and minimize temperature variability, all NIR measurements were conducted at least 10 min after installing the cell in the system. Under these conditions, the cell-surface and internal-water temperatures were nearly equivalent and collectively referred to as "temperature" in the main text.

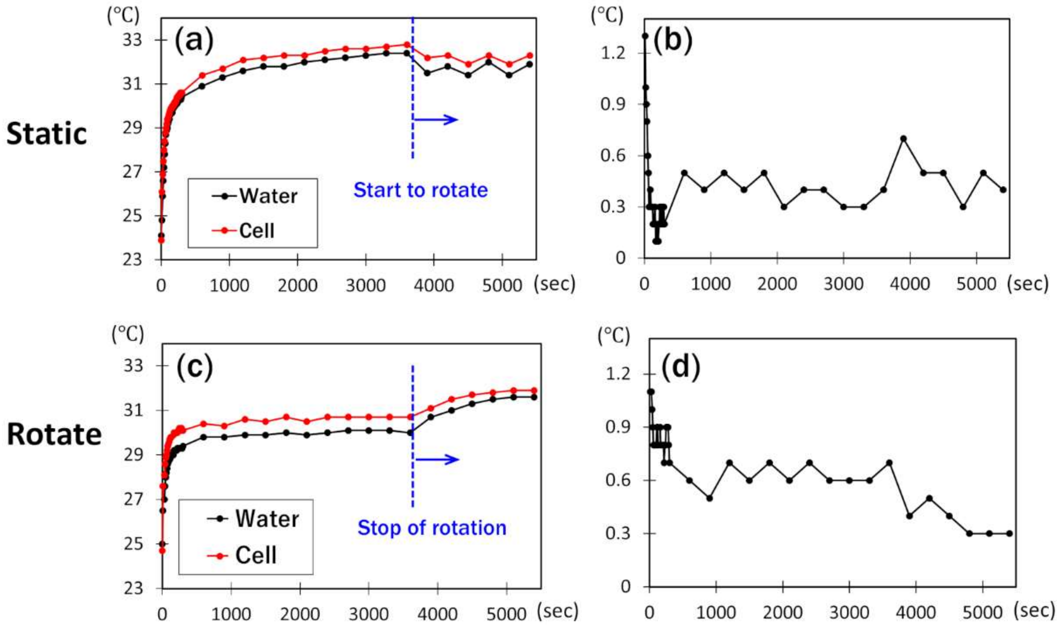

Figure S1: (a) and (c) Temporal variations in both the temperature of water inside the cell (T_{water}) and the temperature at the cell surface (T_{cell}) after cell installation into the NIR system under static (a) and rotating (c) conditions. (b) and (d) Temporal variations in the temperature difference, calculated as $T_{\mathrm{cell}} - T_{\mathrm{water}}$, under static (b) and rotating (d) conditions.

SI 2. Verification of the microgravity environment established by the 3D clinostat

Figure S2 shows an example of measuring the gravitational acceleration acting on a sample placed on the clinostat using a gravitational acceleration sensor (Figure 1b). The horizontal axis represents the elapsed time, while the vertical axis shows the time-averaged gravitational acceleration acting along the x, y, and z axes. As shown in Figure S2, the gravitational acceleration along all three axes converged to within ±0.1 G after approximately 6 to 10 min. Therefore, data in the rotating state were collected only after more than 10 min had passed since the start of rotation.

To examine the effects of rotation on the NIR instrument, background variations were investigated. Figure S3 shows background variations measured under static and rotating conditions, recorded every five minutes for a total of eight measurements. Under the static condition, background variations were suppressed within −0.002, whereas under the rotating condition, variations were observed within ±0.03. However, positive and negative fluctuations occurred randomly with respect to the elapsed time from the start of rotation. The difference in the sign of the background values is considered to be caused by tilting of the instrument during measurement. Furthermore, by increasing the number of trials and averaging the data, these fluctuations were suppressed to a level comparable to the background variation in the static state, indicating that background fluctuations induced by rotation are effectively canceled out through averaging. In addition, no new peaks attributable to rotation were observed. Although baseline variations due to rotation appeared in the principal components obtained by PCA, they were confirmed not to affect peak positions.

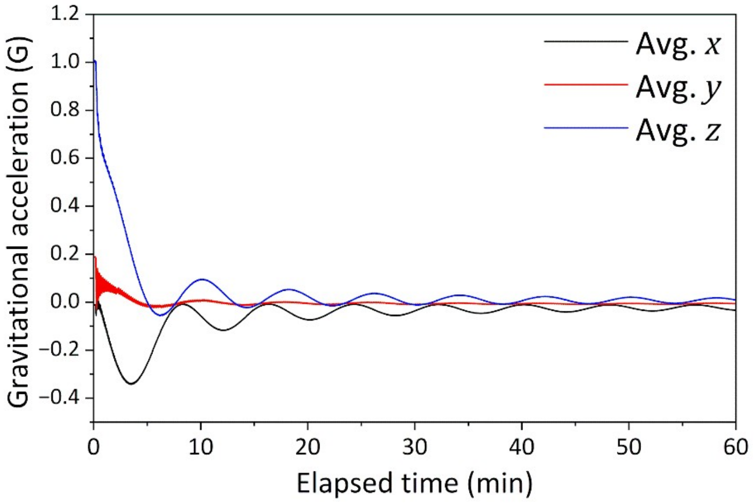

Figure S2: An example of measuring the gravitational acceleration acting on a sample placed on the clinostat using a gravitational acceleration sensor. The sample is rotated around two axes on the clinostat to cancel out the effect of gravity by time averaging.

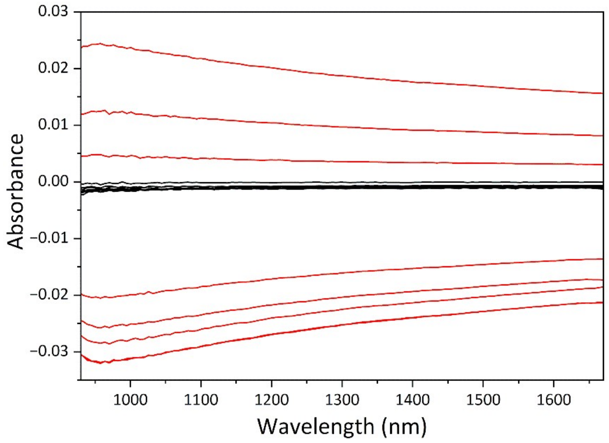

Figure S3: Background variations at the static (black) and rotating (red) states were recorded every five minutes for eight measurements.

SI 3. Detailed discussion of the temperature-dependent NIR band shifts using PCA

In PCA, the first principal component (PC1) is the axis that captures the greatest variance in the data. Subsequent PCs capture the next highest variance under the constraint of orthogonality to previously defined components. The mean-centered spectral data matrix (W) can be approximated as a linear combination of the score vectors (T) and the loading vectors (P), as shown in Eq. (1):

$$W = T_1P_1^T + T_2P_2^T + T_3P_3^T + \cdots \quad (1)$$

The corresponding loading vectors can be interpreted as directions that represent systematic differences between groups in the dataset.

Figure 2a shows NIR spectra in the region of 1000–2500 nm obtained by the benchtop NIR system at temperatures of 10–80 °C with intervals of 5 °C. Increasing the temperature shifted the water bands near 1450 and 1920 nm to shorter wavelengths. As the temperature increased, hydrogen bonds between water molecules tended to break, and the water absorption bands exhibited a blue shift corresponding to changes in the HBN[15–17,20].

Subtraction spectra in the regions of 1300–1700 and 1800–2200 nm (Figure S4) were obtained by subtracting the spectrum at 10 °C as the reference spectrum. With increasing temperature, the contributions of the spectral components with the shorter wavelength (1410 and 1902 nm) increased, whereas those with the longer wavelength (1487 and 1980 nm) decreased. The spectral component whose relative intensity increased at lower temperatures was attributed to SHB water, whereas the component whose relative intensity increased at higher temperatures was attributed to WHB water[15–17,20]. As the temperature increased, the contribution of WHB water increased, whereas that of SHB water decreased, thus explaining the overall blue shift of the water band.

To systematically analyze changes in the absorption bands of water with temperature variation, PCA was performed by focusing on the region of 1000–1670 nm, which contained overlapping peaks from spectra obtained by the benchtop NIR system and the portable NIR system. The PCA score plot (PC1 vs PC2) and PC1 loading plot are shown in Figure 2b. PC1 accounted for 99.7% of the variance and corresponded to the component describing spectral changes associated with temperature variation. In the

PC1 loading plot, a positive peak was observed at 1412 nm and a negative peak at 1486 nm. With increasing temperature, the PC1 score increased monotonically, indicating that the spectral component represented by PC1 loading contributed more strongly to the water spectra at higher temperatures. Because multiplying PC1 loading by the PC1 score reconstructs the mean-centered spectrum, as shown in Equation (1), it can be interpreted that, with increasing temperature, the band intensity at 1412 nm strengthens and that at 1486 nm weakens, resulting in an overall shift of the water band toward a shorter wavelength. Thus, shifts in the spectral band of water corresponding to gravity-induced changes in the HBN of water can be systematically analyzed using PCA.

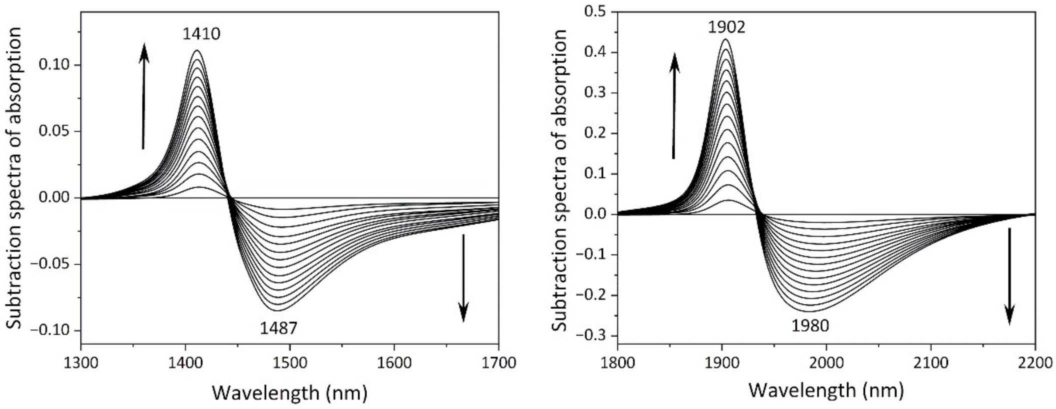

Figure S4: Subtraction spectra in the regions of 1300–1700 and 1800–2200 nm obtained by subtracting the spectrum at 10 °C as the reference spectrum.

SI 4. Supporting figures, table, and statistical analysis for ultrapure water

(a)

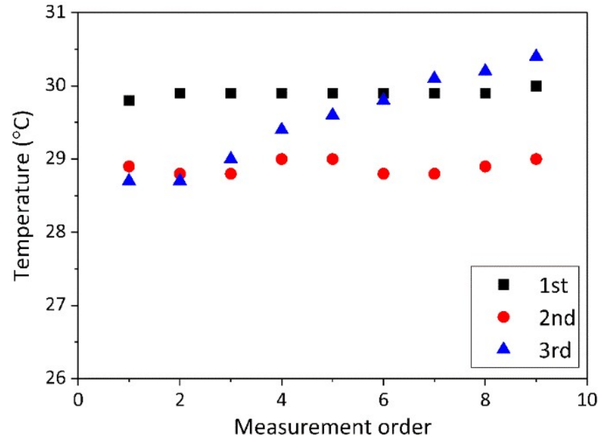

(b)

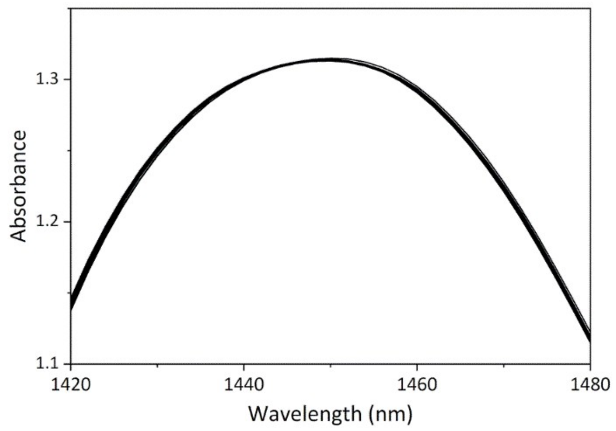

(c)

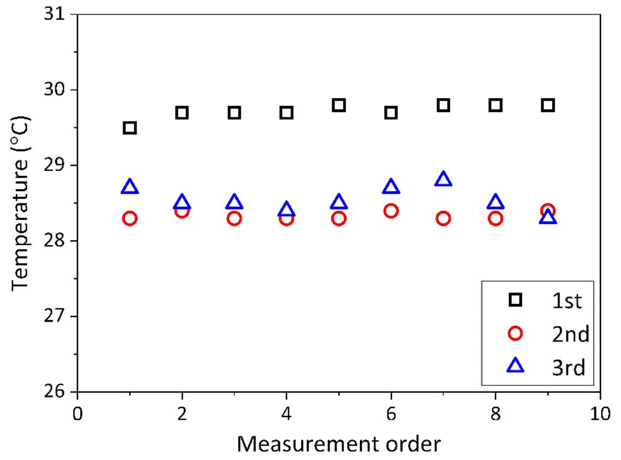

Figure S5: (a) Temperature of ultrapure water recorded every 5 min for 40 min after 20 min of temperature stabilization under static conditions. (b) NIR

spectra of ultrapure water in the region of 1420−1480 nm obtained every 5 min for 40 min after 20 min of temperature stabilization under static conditions. Variation in the temperature during the measurement is expressed as 3rd in Figure S5a. (c) Temperature of ultrapure water recorded every 5 min for 40 min after 20 min of gravity stabilization under rotating conditions.

Table S1: Temperature range divided into 2 °C chosen to reconduct PCA.

Series	Temperature range (°C)
I	29.5−31.5
II	29.0−31.0
III	28.5−30.5
IV	28.0−30.0

Table S2: Summary of *p*-values between static and rotating states calculated for PCA scores in all temperature ranges. Cells with *p*-values less than 10^{-3} are highlighted in gray.

	PC1	PC2	PC3	PC4	PC5
**I**	0.88	1.9×10^{-6}	1.1×10^{-5}	3.1×10^{-3}	7.2×10^{-14}
**II**	0.32	0.38	5.2×10^{-14}	2.0×10^{-2}	6.4×10^{-7}
**III**	0.13	0.29	6.5×10^{-16}	0.83	1.1×10^{-4}
**IV**	0.93	6.4×10^{-3}	4.3×10^{-6}	0.41	2.3×10^{-4}

(a)

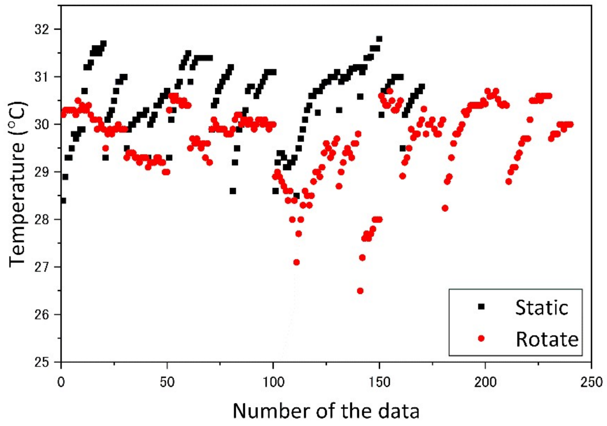

(b)

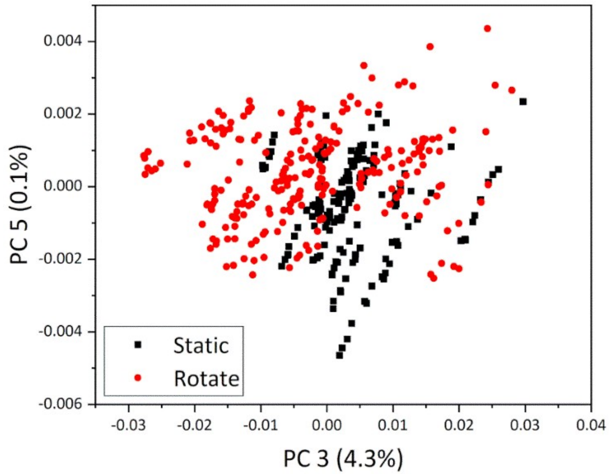

(c)

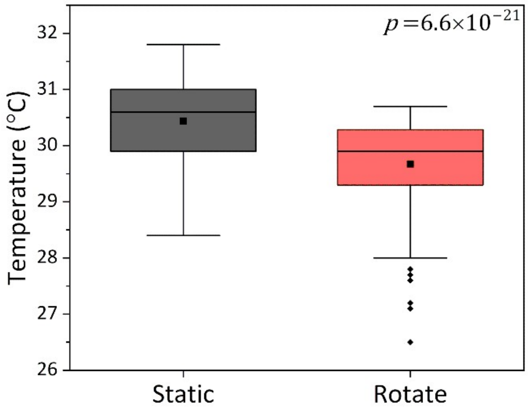

Figure S6: (a) Temperature of ultrapure water obtained every 5 min under static and rotating conditions. (b) Score plots of PCA (PC3 vs PC5) conducted on all NIR spectra acquired under both static and rotating conditions. (c) Box plots of temperature recorded under static and rotating conditions.

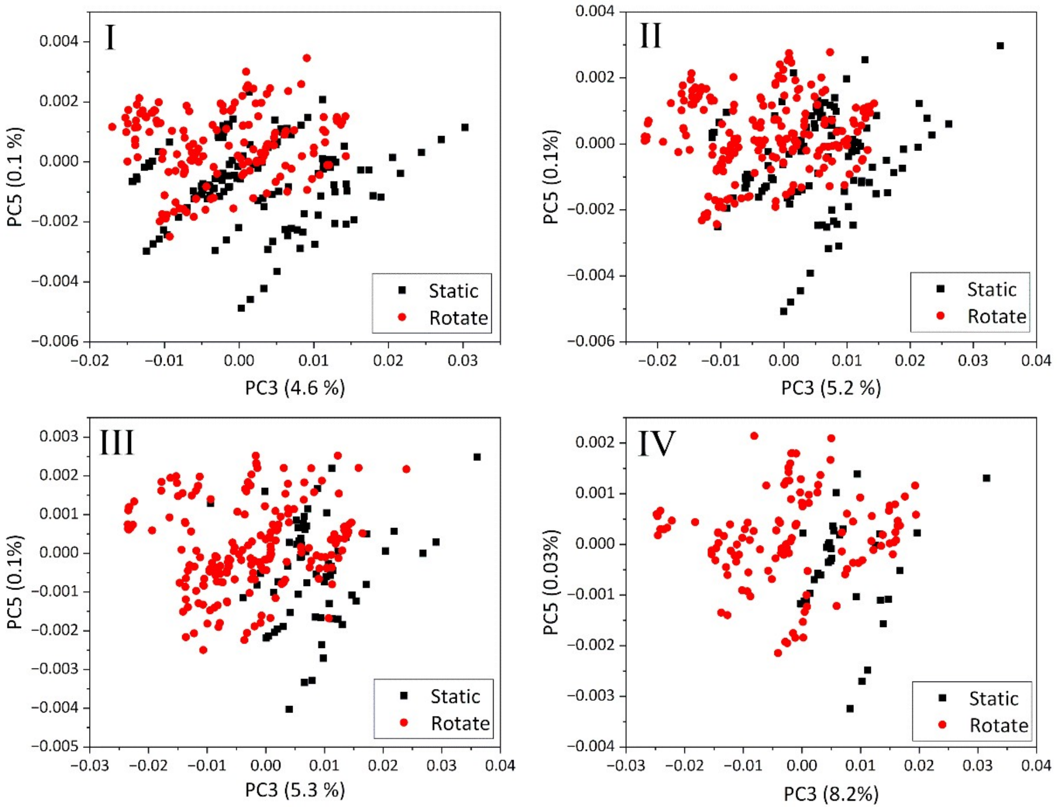

Figure S7: Score plots of PCA (PC3 vs PC5) conducted on water spectral data in all temperature ranges.

SI 5. Supporting figures, table, and statistical analysis for ionic solutions

Tables S3, S4, and S5 summarize the parameters needed to assess the effect of gravity on the HBN of water containing ions. To evaluate the effect of gravity on the HBN of water containing ions (Na_2CO_3, NaCl, NaI, NaSCN), PCA was conducted on the dataset of each solution under the restriction of a temperature range of approximately 2 °C. Scores and loading plots of PCs related to the gravitational function are depicted in Figure S8.

Table S3: Temperature range divided into 2 °C chosen to reconduct PCA on the dataset of the CH_3COONa aqueous solution. The *p*-values were calculated for temperature differences between static and rotating states. Correlation coefficients (CEs) of the relationship between PC3 and gravitational function.

Series	Temperature range (°C)	*p*–value	CE
I	28.0−30.0	4.0×10^{-2}	-0.71
II	27.5−29.5	1.0×10^{-3}	-0.69
III	27.0−29.0	0.65	-0.78
IV	26.5−27.5	0.42	-0.71

Table S4: (A) PC correlating the gravitational function, (B) correlation coefficient between the PC in (A) and gravitational function, and (C) *p*-values for temperature variations between static and rotating states.

	(A)	(B)	(C)
**Na_2CO_3**	PC 3	0.71	0.17
**NaCl**	PC 2	0.62	0.19
**NaI**	PC 1	0.78	0.28
**NaSCN**	PC 1	0.90	0.65

Table S5: Correlation coefficient of the relationship between PC3 scores and gravitational function.

	Na_2CO_3	CH_3COONa	NaCl	NaI	NaSCN
**CE**	–0.07	0.16	–0.59	–0.91	–0.63

Na_2CO_3

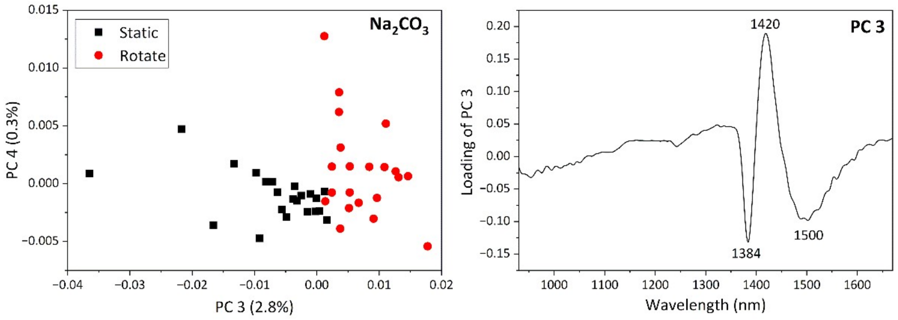

NaCl

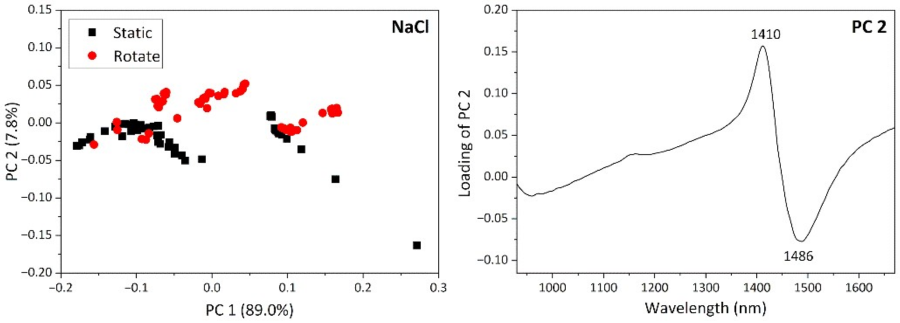

NaI

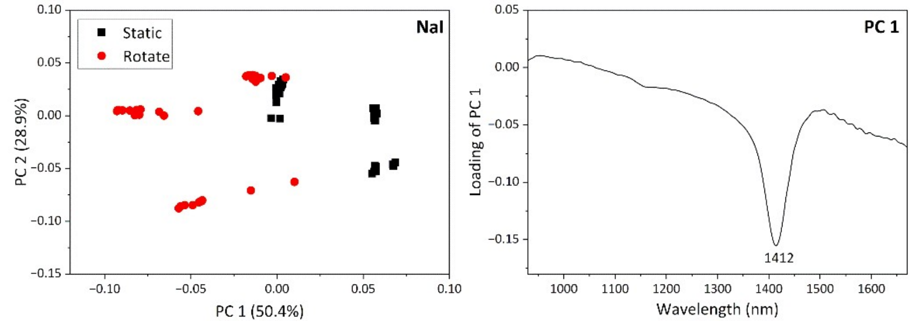

NaSCN

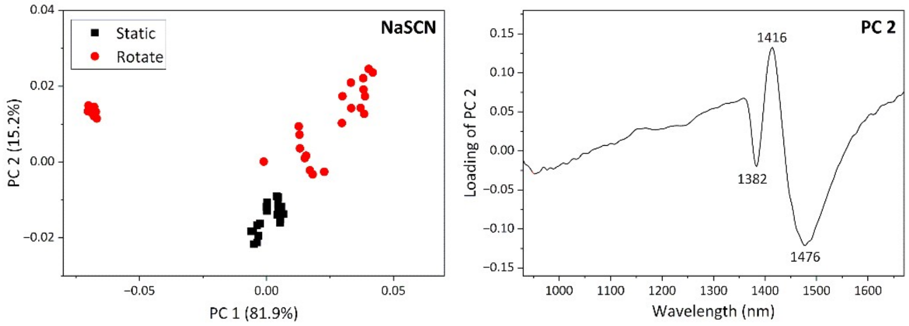

Figure S8: Score and loading plots of PCA conducted on the dataset of each aqueous solution containing one of five species of inorganic salts (Na_2CO_3, NaCl, NaI, NaSCN) obtained under both static and rotating conditions.
